# Eating Disorder Symptoms in the Context of Perfectionism and Sociocultural Internalization: A Profile Analysis and Mediation Approach

**DOI:** 10.3390/nu18010161

**Published:** 2026-01-03

**Authors:** Karolina Szymajda, Magdalena Chęć, Sylwia Michałowska

**Affiliations:** 1 Eurospec School in Szczecin, 47 Adama Mickiewicza St., 70-385 Szczecin, Poland; 2 Department of Clinical Psychology and Psychoprophylaxis, Institute of Psychology, University of Szczecin, 69 Krakowska Street, 71-017 Szczecin, Poland; sylwia.michalowska@usz.edu.pl

**Keywords:** eating disorders, perfectionism, sociocultural internalization, latent profile analysis, mediation analysis

## Abstract

**Background**: This study examined the interplay between sociocultural internalization, perfectionism, and eating disorder (ED) symptoms. We pursued two complementary aims: (1) to identify latent profiles of women based on adaptive/maladaptive perfectionism and sociocultural internalization, and (2) to test perfectionism as a mediator between sociocultural internalization and ED symptoms. **Methods**: Participants comprised 203 Polish women aged 18–35 years (M = 25.1, SD = 3.5). Measures included the Eating Attitudes Test-26 (EAT-26), the Polish Adaptive and Maladaptive Perfectionism Questionnaire (KPAD), and the Sociocultural Attitudes Towards Appearance Questionnaire-3 (SATAQ-3). Latent profile analysis (LPA) was used to identify subgroups, followed by Kruskal–Wallis tests for between-profile comparisons. Mediation models were tested using the PROCESS macro (Model 4). **Results**: A three-profile solution provided the best fit (Entropy = 0.94). Profile 3 (high internalization and both perfectionism types; *n* = 58) reported the highest ED severity (EAT-26 total: M = 25.6, SD = 7.4), particularly in Dieting and Bulimia subscales. Profile 1 (low internalization, low maladaptive perfectionism; *n* = 64) showed the lowest scores (M = 12.3, SD = 5.2). No significant differences were found for the Oral Control subscale (H(2) = 2.53, *p* = 0.283). Mediation analyses indicated that maladaptive perfectionism significantly mediated associations between sociocultural internalization and ED symptoms (indirect effects b = 0.13–0.32, 95% CI excluding zero). Adaptive perfectionism was not a significant mediator. **Conclusions**: Results underscore maladaptive perfectionism as a key mechanism through which sociocultural pressures contribute to eating pathology. Implications include targeting internalization and perfectionistic concerns in prevention and treatment.

## 1. Introduction

The etiology of eating disorders (ED) is complex and involves the interaction of multiple risk factors, including personality traits and cultural influences [1]. The literature particularly emphasizes the role of sociocultural internalization and perfectionism, which are considered important predictors of the development of eating disorders [2,3].

Sociocultural internalization refers to the degree to which individuals adopt social appearance ideals as their own standards [4]. These norms are communicated through various social and cultural channels. These standards may be difficult to achieve. [5,6]. Studies show that the internalization of such messages, along with accompanying social pressure, fosters excessive comparison with others, which is linked to body dissatisfaction, low self-esteem, and disordered eating behaviors [7,8]. This phenomenon is especially pronounced in Western culture, where the slim body ideal remains strongly entrenched and widely promoted, thereby intensifying the drive to meet unattainable standards [9,10].

Another risk factor is perfectionism, which—according to Hamachek’s [11] dual model—can be adaptive or maladaptive. Adaptive perfectionism may foster achievement and self-improvement, whereas maladaptive perfectionism is linked to excessive concern about mistakes, setting unrealistic demands, and fear of negative evaluation. Numerous studies have associated maladaptive perfectionism with eating disorder symptoms, such as dietary restriction, body dissatisfaction, and perceived lack of control [12,13,14,15]. Maladaptive perfectionism may increase vulnerability to psychological distress and disordered eating behaviors [16,17,18]. Importantly, research suggests that maladaptive perfectionism not only functions as a direct risk factor but also as a mediating mechanism linking other psychological variables to eating disorder symptoms. For instance, socially prescribed perfectionism mediated the relationship between cultural influences and the severity of muscle dysmorphia and eating disorders [19]. Another study demonstrated that perceived criticism, through maladaptive perfectionism, was linked to an increased drive for thinness [20]. Lower body self-esteem and higher BMI have been associated with increased risk of developing eating disorders [21]. These results highlight the need for early identification of risk factors and preventive interventions targeted at the most vulnerable groups, especially young women.

Previous research has mainly focused on direct associations between perfectionism and eating disorder (ED) symptoms, but less is known about its mediating role in the relationship between sociocultural internalization and symptom severity. Few studies have applied person-centered approaches, such as latent profile analysis (LPA), to identify subgroups with distinct combinations of internalization and perfectionism, and the mediating role of maladaptive or adaptive perfectionism within these profiles remains underexplored.

Combining LPA with mediation analysis allows for the identification of high-risk subgroups while simultaneously clarifying the mechanisms linking internalization and perfectionism to ED risk. This integrated approach provides a more nuanced understanding of who is at risk and why, informing targeted preventive and therapeutic interventions.

Accordingly, we formulated the following hypotheses:

**H1.**  *Distinct profiles of young women can be identified based on the dimensions of sociocultural internalization and the two types of perfectionism, which differ in the levels of these characteristics.*

**H2.**  *Profiles characterized by higher sociocultural internalization and higher levels of perfectionism—particularly maladaptive perfectionism—will exhibit greater severity of eating disorder symptoms compared to profiles with lower levels of these characteristics.*

**H3.**  *Maladaptive perfectionism positively mediates the relationship between sociocultural internalization and eating disorder symptoms.*

**Figure 1:**
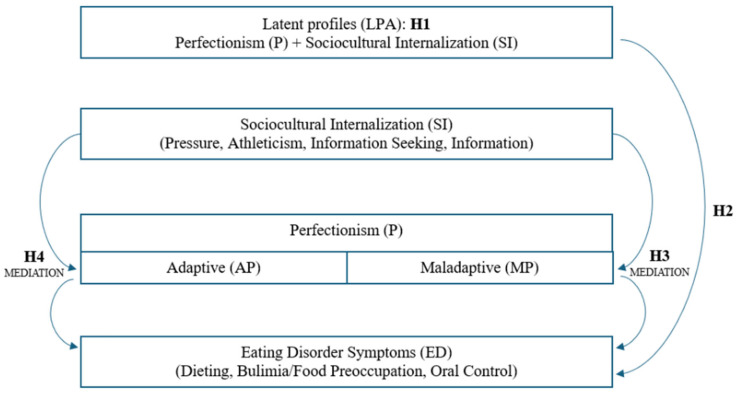
Conceptual model of the study presenting hypothesized relationships between sociocultural internalization, perfectionism, and eating disorder symptoms, including latent profiles (H1–H4).

**H4.**  *Adaptive perfectionism positively mediates the relationship between sociocultural internalization and eating disorder symptoms, although this effect is expected to be weaker than the mediation through maladaptive perfectionism (Figure 1).*

## 2. Materials and Methods

### 2.1. Participants

**Figure 2:**
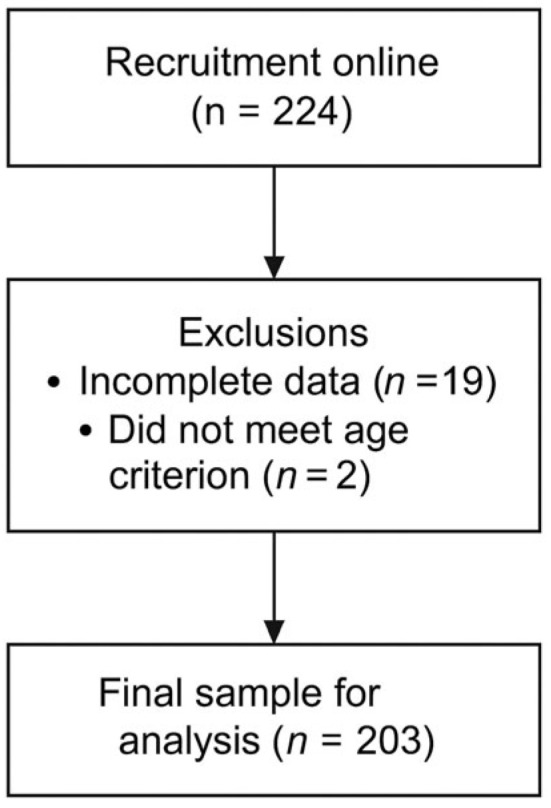
Flowchart of the recruitment and sample selection process.

The study included 203 women of Polish nationality. The inclusion criterion was age between 18 and 35 years. The mean age of the participants was 25.08 years (SD = 3.50). Figure 2 presents the flowchart of the recruitment procedure and sample selection.

The majority of the sample consisted of individuals with higher education or those currently enrolled in university studies (*n* = 85). Eighty-four participants were employed, and 106 reported being in a romantic relationship. In addition, 60 participants lived in cities with more than 500,000 inhabitants. Almost all participants (*n* = 201) used social media. The BMI of the studied group ranged from 16.01 to 35.62, covering underweight, normal weight, overweight, and obesity. The characteristics of the study participants are presented in Table 1.

The demographic variables included in this study (age, BMI, education, relationship status, and place of residence) were selected based on prior research showing that these factors differentiate levels of eating disorder risk. Age and BMI have been linked to dieting behaviors and body dissatisfaction [6,22]. Educational level and urban residence are associated with greater exposure to sociocultural appearance pressures [23]. Relationship status has also been shown to relate to differences in appearance-related anxiety [21]. Therefore, these demographics were collected to appropriately characterize the sample and ensure comparability with previous research.

### 2.2. Procedure

The study was conducted between October 2024 and the end of January 2025 in an online format using the LimeSurvey platform (version 5.7 or the version current at the time of data collection). Ethical approval was obtained from the appropriate ethics committee. Participants were recruited via multiple online channels, including social media platforms (e.g., Facebook, Instagram), online interest groups, and discussion forums relevant to young adults and health/psychology topics. The survey link was posted publicly in these groups, and individuals were invited to participate voluntarily. All participants were provided with information about the purpose of the study, the voluntary nature of participation, the anonymity and confidentiality of responses, and the possibility to withdraw at any point without consequence.

As participation was self-selected, the sample may be subject to selection bias. Individuals with a particular interest in eating behaviors, body image, or psychological research may have been more likely to participate, and some demographic groups may have been underrepresented. Additionally, the online recruitment method relies on access to the internet and familiarity with the platforms used, which could limit the generalizability of the findings.

### 2.3. Measures

#### 2.3.1. Eating Disorder Risk

Eating disorder risk was assessed using the Eating Attitudes Test-26 (EAT-26) developed by Garner et al. [24] in the Polish adaptation by Rogoza, Brytek-Matera, and Garner [25]. The test includes a general scale and three subscales: Dieting, Bulimia and Food Preoccupation, and Oral Control. The Dieting subscale relates to behaviors associated with dietary restriction and fear of weight gain; the Bulimia and Food Preoccupation subscale concerns uncontrolled binge eating, compensatory behaviors, and obsessive thoughts about food, while the Oral Control subscale captures the sense of control over food and appetite monitoring.

Scores can range from 0 to 78. A score of 20 or more indicates risk of an eating disorder and the need for professional diagnostic assessment. Higher scores reflect greater symptom severity. The global reliability of the scale is α = 0.80, and its validity has been confirmed through correlations with other measures of eating pathology.

#### 2.3.2. Perfectionism

Perfectionism was measured with the Polish Adaptive and Maladaptive Perfectionism Questionnaire (KPAD) by Szczucka [26]. The KPAD assesses two types of perfectionism: adaptive perfectionism (AP) and maladaptive perfectionism (MP). Reliability is α = 0.92 (AP) and α = 0.95 (MP). Theoretical validity has been confirmed through correlations with conscientiousness (AP: r = 0.472), self-handicapping (MP: r = 0.342), and neuroticism (MP: r = 0.729). Higher scores indicate stronger levels of the respective type of perfectionism.

#### 2.3.3. Sociocultural Internalization

Sociocultural internalization was assessed with the Sociocultural Attitudes Toward Appearance Questionnaire-3 (SATAQ-3) [27], in the Polish adaptation by Izydorczyk and Lizińczyk [28]. The questionnaire contains subscales: Pressure–Internalization, Information Seeking–Internalization, Internalization–Athleticism, and Information. The Pressure–Internalization subscale consists of 12 items describing the level of internalization of sociocultural appearance norms resulting from perceived media pressure. Information Seeking–Internalization subscale includes 6 items describing internalization related to seeking body image information. The Internalization–Athleticism subscale has 4 items assessing the internalization of norms emphasizing a fit and athletic body. The Information subscale includes 6 items measuring the frequency of seeking body image-related information in mass media. High scores in each scale indicate greater sociocultural influence and internalization of appearance norms. The reliability of subscales ranged between α = 0.76–0.92.

#### 2.3.4. Demographics

Additionally, a custom questionnaire was used to collect demographic data, including age, biological sex and gender identity, education, employment status, place of residence, marital status, height, weight (for BMI calculation), and social media usage habits.

### 2.4. Data Analysis

Statistical analyses were performed using IBM SPSS Statistics 29.0 (IBM Corporation, Armonk, NY, USA) and Jamovi 2.4.11 (The Jamovi Project, Sydney, NSW, Australia).

To identify groups of participants based on internalization dimensions and perfectionism, latent profile analysis (LPA) was conducted. The total sample consisted of 203 participants. Although moderate, this sample size aligns with recommended thresholds for latent profile analysis outlined in the literature. Research by Spurk et al. [29] suggests that samples around 200 can provide stable profile solutions, depending on model complexity and the number of indicators. Tein et al. [30] also support similar sample size ranges for acceptable power to detect correct class enumeration. We advise cautious interpretation of smaller profiles due to reduced group size, but the overall sample should be considered adequate for exploratory LPA.

The latent profile analysis (LPA) was conducted using the SnowRMM module in Jamovi. The analysis utilized the default maximum likelihood estimator available within the module. Convergence criteria were based on the module’s internal iterative algorithm, which continues until parameter estimates stabilize below a preset tolerance level; however, precise convergence thresholds are not explicitly reported in the output. The model estimation incorporated multiple random starts (typically between 50 and 100) to avoid local maxima, though the exact number of random starts used is set to Jamovi defaults and may not be directly accessible or modifiable via the interface. For model selection, fit indices such as the Bayesian Information Criterion (BIC), Akaike Information Criterion (AIC), and entropy were examined to identify the optimal number of latent profiles. Posterior probabilities and classification accuracy metrics were also evaluated to ensure good profile separation. Due to limitations in the SnowRMM module’s reporting capabilities, detailed technical specifications such as exact convergence criteria values and estimation algorithm parameters cannot be provided. Therefore, the analysis adhered to the default settings as implemented in Jamovi’s SnowRMM module, which are consistent with standard practices for latent profile modeling.

Group comparisons were made using the Kruskal–Wallis H test. Dunn’s post hoc test with Bonferroni correction was applied. The mediating role of perfectionism in the relationship between sociocultural internalization and eating disorder risk was tested using Hayes’ PROCESS macro (Model 4). The significance level was set at α = 0.05. Bootstrap was applied with 5000 samples, and the variables were not standardized.

Results are presented in three stages: (1) latent profile analysis; (2) group comparisons on eating disorder symptoms; and (3) mediation analyses to determine the role of perfectionism in linking internalization to eating pathology.

BMI was not included as a covariate because it did not differ between profiles (*p* = 0.350) and showed no significant correlations with the key variables. Consistent with methodological guidance, covariates should be included only when theoretically and empirically justified [31,32].

## 3. Results

### 3.1. Latent Profile Analysis

For the dimensions of perfectionism (adaptive and maladaptive) and internalization (pressure, athleticism, information seeking, and information), a latent profile analysis was performed. This method allows the identification of hidden groups within the dataset that are characterized by similar response patterns on the analyzed variables.

**Figure 3:**
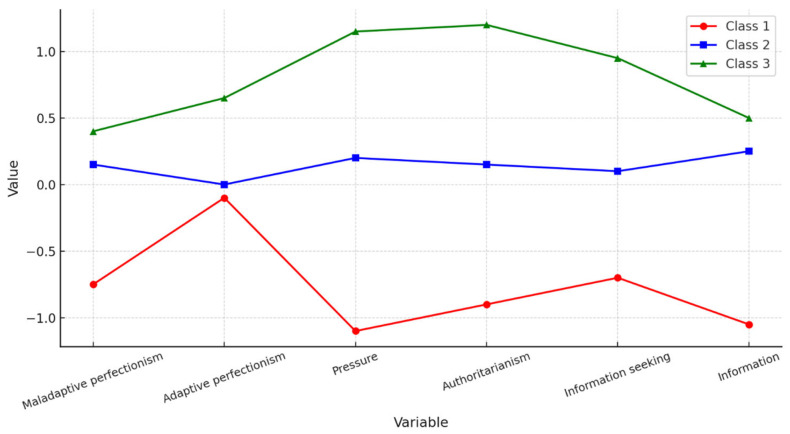
Latent Profiles.

Based on model fit indices (Table 2), the solution with the best fit was a model assuming varied variances and covariances in each identified class, with the highest entropy value. Specifically, Model 6 with three classes (profiles) proved to be optimal: AIC = 2901, BIC = 3176; Entropy = 0.943 (Figure 3).

Comparative analysis of the identified participant profiles showed statistically significant differences for all perfectionism and internalization dimensions analyzed (Table 3). Means (M) and Standard Deviations (SD) for Perfectionism and Internalization Variables Across Three Profiles are presented in Table 4.

Detailed post hoc analysis indicated that the level of maladaptive perfectionism among participants in Profile 2 and Profile 3 was similar (*p* > 0.05), whereas in Profile 1 it was significantly lower than in the other two groups (*p* < 0.001). Adaptive perfectionism was highest among individuals in Profile 3 and significantly higher than in the other groups. No differences were noted between Profiles 1 and 2.

For the internalization dimensions: pressure, athleticism, and information seeking, differences were found between all groups, with the highest results in Profile 3 and the lowest in Profile 1. For the Information scale, a significantly lower level was found in Profile 1 compared to the other two groups, while Profiles 2 and 3 did not differ.

### 3.2. Profiles and Eating Disorders

The Kruskal–Wallis H test was used to compare the identified profiles in terms of EAT-26 dimensions (Table 5). The analysis showed that the groups differed in terms of overall eating disorder severity, as well as the Dieting and Bulimia/Food Preoccupation subscales. No statistically significant differences between groups were observed for the Oral Control subscale.

**Figure 4:**
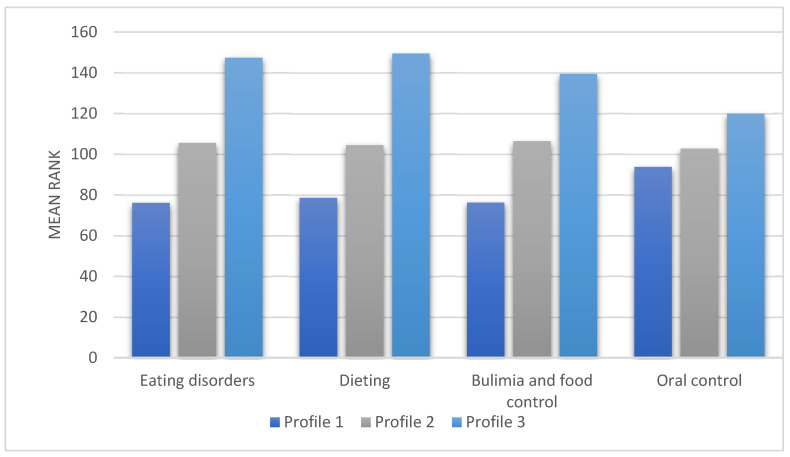
Mean ranks for eating disorder dimensions in identified profiles.

Post hoc analysis showed that individuals in Profile 3 had higher levels of eating disorder symptoms, dieting, and bulimia/food preoccupation than individuals in the other groups (*p* < 0.05). In addition, individuals in Profile 2 demonstrated higher eating disorder severity and dieting compared to individuals in Profile 1. For bulimia/food preoccupation. No differences were observed between Profiles 1 and 2 (*p* > 0.05; Figure 4).

### 3.3. Mediating Role of Perfectionism

Table 6 presents a summary of the results of mediation models that included the mediating role of maladaptive perfectionism in the relationship between sociocultural internalization and ED risk. The analysis confirmed the mediating effect of perfectionism in all tested models. In Models 1–3, partial mediation was observed, whereas in Model 4 full mediation was confirmed.

The results indicated that each of the sociocultural internalization dimensions (pressure, athleticism, information seeking, and information) was associated with higher maladaptive perfectionism, and higher perfectionism in turn was associated with greater eating disorder severity, although the size of these associations reflected a small effect.

Analogous analyses were performed for adaptive perfectionism. These analyses did not confirm the mediating role of this variable in the relationship between sociocultural internalization and eating disorders (Table 7).

In the case of three internalization dimensions (pressure, athleticism, and information seeking), partial mediation was observed, meaning that maladaptive perfectionism explained part, but not the whole relationship between internalization and symptom severity and the magnitude of these indirect effects was small. For the “Information” dimension, full mediation was observed, indicating that the relationship between internalization and eating disorder symptoms occurred entirely through maladaptive perfectionism.

## 4. Discussion

The aim of the present study was to analyze the relationships between dimensions of perfectionism and internalization and the severity of eating disorders among the participants. The latent profile analysis distinguished three groups that differed in their level of sociocultural norm internalization and types of perfectionism. The obtained results allow for an in-depth interpretation of risk mechanisms in the context of eating disorders.

Profile 1 showed low susceptibility to social and internal pressures, Profile 2 exhibited moderate levels, and Profile 3 demonstrated the highest levels of internalization and perfectionism, indicating increased vulnerability to cultural norms and self-imposed demands.

The results show that higher levels of adaptive and maladaptive perfectionism co-occur with stronger internalization of sociocultural pressures, drive for athleticism, and seeking appearance-related information in the media. Individuals in Profile 3 were more inclined toward self-control and internalization of cultural norms, which is consistent with earlier studies suggesting that maladaptive perfectionism is associated with internal pressure and higher severity of eating disorder symptoms [16,18]. According to the meta-analysis by Stackpole and colleagues [33], both perfectionistic striving and perfectionistic concerns are associated with eating disorder symptoms in adults, in both clinical and non-clinical samples. The current study revealed a similar pattern: adaptive perfectionism was linked with moderate risk, whereas maladaptive perfectionism demonstrated stronger associations with symptom severity.

Studies conducted in Poland [23] showed that internalization of sociocultural appearance norms is a significant predictor of drive for thinness and body dissatisfaction, particularly among younger girls. These findings align with previous studies indicating that higher internalization and perfectionistic concerns are associated with increased eating disorder risk [34]. This highlights the importance of both individual and environmental factors in prevention and intervention. The study by Habashy and Culbert [35] further demonstrated that maladaptive perfectionism is significantly associated with eating disorders, and that pressure for thinness and internalization of the thin ideal fully mediates this relationship. The present findings support these observations, emphasizing the role of internal pressure and cultural norm internalization as factors linking perfectionism with eating disorder risk.

Mediation analyses confirmed that maladaptive perfectionism mediates the relationship between internalization and eating disorder severity, highlighting its role as a key vulnerability factor. This indicates that internalization of media messages and social pressure is associated with higher levels of maladaptive perfectionism, which in turn is linked to greater severity of eating disorder symptoms. This finding is consistent with theoretical concepts proposing that maladaptive perfectionism acts as a vulnerability factor, strengthening the negative impact of cultural pressure on self-image and eating behaviors [35].

In contrast, analyses involving adaptive perfectionism did not confirm its mediating role in the examined relationships. This result suggests that although some forms of adaptive perfectionism may be associated with moderate risk, they do not constitute a significant mechanism linking internalization with eating disorders. It can be assumed that striving for high standards alone is not necessarily associated with dysfunctional behaviors unless accompanied by the critical and anxious components typical of maladaptive perfectionism. These findings highlight the importance of maladaptive perfectionism as a key element linking sociocultural influence with individual vulnerability to eating disorders. In practice, this means that preventive and therapeutic interventions should focus not only on reducing social pressure and loosening cultural norms, but also on addressing internal perfectionistic mechanisms, especially maladaptive ones.

Profile 3 exhibited the highest severity of eating disorder symptoms, Profile 2 showed moderate severity, and Profile 1 the lowest, indicating that high internalization and maladaptive perfectionism are key risk factors. The lack of significant differences regarding Oral Control, i.e., the need to control eating and the effort invested in suppressing appetite, indicates that not every aspect of eating behavior is a direct consequence of personality traits (perfectionism) or socio-cognitive processes (internalization of norms). Some may instead depend on more general biopsychosocial mechanisms. A study conducted among Greek dietetics students found a positive correlation between Oral Control and men’s BMI [22], which may suggest its association with biological factors (e.g., hunger and satiety regulation, genetics, hormones) or with social norms prevalent in a given culture or social group (e.g., pressure for “fit eating”), which apply universally and not only to individuals with higher perfectionism.

The results support the cognitive–behavioral model of eating disorders [36], in which self-criticism and high self-expectations are key risk factors. From the perspective of clinical practice and prevention, these findings indicate the need to individualize interventions, taking into account both the level of cultural norm internalization and the dominant type of perfectionism. Intervention strategies should focus on reducing internal pressure, fostering adaptive perfectionism, and shaping realistic self-standards, which may reduce vulnerability to restrictive eating behaviors and weight control disturbances.

An important contribution of this study is the combined use of latent profile analysis and mediation models to identify subgroups of young adults characterized by distinct patterns of co-occurrence between internalization and different dimensions of perfectionism, and to examine how these patterns relate to eating disorder severity. This approach allows for a more nuanced understanding of heterogeneity in risk factors and informs the development of tailored preventive and therapeutic interventions.

Future research should not only continue analyzing the interactions between perfectionism and sociocultural internalization but also incorporate new approaches, such as network models or longitudinal analyses, which could capture dynamic dependencies and changes over time in the development of eating disorders.

### 4.1. Limitations

The study has several limitations. First, the research sample was not evenly distributed across the identified profiles, particularly Profile 3, which comprised a relatively small number of participants. This may affect the stability and generalizability of the results in comparative analyses. Second, cross-sectional design prevents determination of the direction of relationships and limits causal inferences regarding internalization, perfectionism, and eating disorder severity. Third, participants were not clinically diagnosed, so the findings pertain to symptom severity and risk rather than confirmed eating disorders. Fourth, the use of a self-selected, online sample may have introduced selection bias. Fifth, the reliance on self-report questionnaires carries the risk of reporting errors, and other potential confounding variables, such as stress levels, depressive symptoms, family history, or physical activity, were not measured and may have influenced the observed relationships. Future research should address these limitations by employing clinically assessed samples, accounting for potential confounders, and using longitudinal designs to better establish causal relationships.

### 4.2. Conclusions

Maladaptive perfectionism and sociocultural internalization are key factors associated with increased severity of eating disorder symptoms. High levels of both confer the greatest vulnerability, while adaptive perfectionism does not play a mediating role. These findings support cognitive–behavioral models emphasizing self-criticism and high self-expectations. Interventions should target maladaptive perfectionistic tendencies and challenge internalized cultural pressures. Limitations include the predominantly female Polish sample, small profile sizes, and the cross-sectional design.

## 5. Implications for Practice

The profiles identified in this study indicate that the level of internalization of cultural norms and the type of perfectionism determine the degree of risk for eating disorders. Profiling enables the differentiation of high- and low-risk groups, which have direct applications in clinical practice and prevention. For practitioners, including psychologists, dietitians, and health educators, the findings suggest the necessity of individualizing interventions. Individuals exhibiting high levels of maladaptive perfectionism and strong internalization of social norms (Profile 3) require particular monitoring and support. Targeted interventions may include:

1. Psychoeducational programs aimed at developing critical thinking regarding media messages and cultural norms;
2. Strategies to strengthen psychological resilience and promote a realistic body image;
3. Therapeutic interventions addressing maladaptive perfectionism mechanisms, such as fear of evaluation and excessively high self-imposed standards.

Profiling also allows for preventive efforts among young women through the early identification of high-risk individuals, which may enhance the effectiveness of educational and health promotion programs. At a policy level, the findings may inform the development of strategies for monitoring the impact of social media and implementing tailored psychological support within educational settings.

In summary, classifying participants according to internalization of norms and perfectionism not only deepens the understanding of risk mechanisms for eating disorders but also provides concrete guidance for practitioners regarding prevention, monitoring, and the individualization of therapeutic interventions.

In addition, the findings highlight specific cognitive–behavioral processes that should be targeted in interventions. These include maladaptive perfectionistic thinking, excessive self-criticism, automatic negative thoughts about body image, compulsive comparison with others, and dysfunctional emotion regulation strategies such as restrictive eating or binging. Clinical interventions could focus on cognitive restructuring to challenge distorted beliefs, behavioral experiments to reduce maladaptive eating behaviors, mindfulness and body-acceptance strategies, and resilience training to reduce the impact of social comparisons. Integrating these approaches may enhance the effectiveness of preventive and therapeutic programs, particularly for high-risk individuals identified through profiling.

## Figures and Tables

**Table 1:** Characteristics of the study participants.

Variables	Statistic
Age, M (SD)	25.09 (3.52)
BMI, M (SD)	22.18 (5.14)
Height, M (SD)	1.66 (0.07)
Body weight, M (SD)	61.58 (15.38)
Education, *n* (%)	
Primary education	3 (1.5%)
Basic vocational education	2 (1.0%)
Secondary education	36 (17.7%)
Currently pursuing higher education	85 (41.9%)
Higher education	77 (37.9%)
Employment status, *n* (%)	
Employed	84 (41.4%)
Unemployed	2 (1.0%)
Student	55 (27.1%)
Student and employed	61 (30.0%)
Missing data	1 (0.5%)
Place of residence, *n* (%)	
Rural area	24 (11.8%)
City up to 100.000 inhabitants	44 (21.7%)
City from 100.000 to 250.000 inhabitants	31 (15.3%)
City from 250.000 to 500.000 inhabitants	44 (21.7%)
City over 500.000 inhabitants	60 (29.6%)
Marital status, *n* (%)	
Single	59 (29.1%)
In an informal relationship	106 (52.2%)
In a formal relationship/married	35 (17.2%)
Other	3 (1.5%)

**Table 2:** Fit indices for LPA models.

Model	Classes	LogLik	AIC	AWE	BIC	CAIC	CLC	KIC	SABIC	ICL	Entropy
1	2	−1580	3199	3418	3262	3281	3162	3221	3201	−3284	0.837
1	3	−1530	3112	3413	3198	3224	3062	3141	3116	−3228	0.859
2	2	−1545	3141	3430	3224	3249	3093	3169	3144	−3237	0.890
2	3	−1524	3123	3563	3249	3287	3049	3164	3129	−3271	0.903
3	2	−1490	3048	3442	3161	3195	2982	3085	3053	−3192	0.783
3	3	−1479	3040	3515	3176	3217	2960	3084	3046	−3226	0.767
6	2	−1404	2917	3555	3099	3154	2809	2975	2925	−3106	0.932
6	3	−1367	2901	3864	3176	3259	2737	2987	2913	−3185	0.943

Source: authors’ own study.

**Table 3:** Comparison of profiles in terms of perfectionism and internalization.

	Profile1 (*n* = 47)			Profile 2 (*n* = 140)			Profile 3 (*n* = 16)						Post Hoc
Dependent Variable	Mean Rank	Mdn	IQR	Mean Rank	Mdn	IQR	Mean Rank	Mdn	IQR	H(2)	*p*	η^2^	
Maladaptive perfectionism	62.51	55.00	51.00	112.76	93.50	43.75	123.88	92.00	39.75	28.16	<0.001	0.13	1–2 *p* < 0.001; 1–3 *p* = 0.001
Adaptive perfectionism	96.40	64.00	16.00	98.56	65.50	19.00	148.53	74.00	9.75	10.95	0.004	0.04	1–3 *p* = 0.006 2–3 *p* = 0.004
Internalization—Pressure	30.87	14.00	6.00	118.61	33.00	19.00	165.63	45.50	8.50	99.04	<0.001	0.49	1–2 *p* < 0.001 1–3 *p* < 0.001 2–3 *p* = 0.007
Internalization—Athleticism	54.95	7.00	7.00	109.51	13.00	6.00	174.53	18.00	3.50	57.08	<0.001	0.28	1–2–3 *p* < 0.001
Internalization—Information Seeking	61.79	10.00	9.00	108.86	17.00	7.00	160.06	22.00	3.50	39.70	<0.001	0.19	1–2 *p* < 0.001 1–3 *p* < 0.001 2–3 *p* = 0.003
Internalization—Information	29.64	6.00	0.00	122.16	13.00	8.00	138.16	15.00	4.00	95.04	<0.001	0.47	1–2 *p* < 0.001 1–3 *p* < 0.001

Note. Mdn = median; IQR = interquartile range; H(2) = Kruskal–Wallis H statistic with 2 degrees of freedom; *p* = significance level; η^2^ = effect size. Profile 1 = low internalization and perfectionism; Profile 2 = moderate internalization and perfectionism; Profile 3 = high internalization and perfectionism.

**Table 4:** Means (M) and Standard Deviations (SD) for Perfectionism and Internalization Variables Across Three Profiles.

	1		2		3	
	M	SD	M	SD	M	SD
Maladaptive perfectionism	65.02	33.34	94.16	27.49	100.44	30.79
Adaptive perfectionism	63.43	15.10	64.42	13.68	74.25	5.89
Internalization—Pressure	15.04	3.28	34.27	12.18	46.19	8.50
Internalization—Athleticism	8.45	4.06	13.07	4.21	18.06	1.57
Internalization—Information Seeking	12.45	6.65	17.18	4.96	21.94	3.40
Internalization—Information	6.23	0.43	14.02	5.63	15.00	3.85

**Table 5:** Comparison of profiles in terms of eating disorders.

	Profile 1 (*n* = 47)			Profile 2 (*n* = 140)			Profile 3 (*n* = 16)						Post Hoc
Dependent Variable	Mean Rank	Mdn	IQR	Mean Rank	Mdn	IQR	Mean Rank	Mdn	IQR	H(2)	*p*	η^2^	
ED	76.13	9.00	10.00	105.51	13.50	12.00	147.31	20.50	15.50	19.18	<0.001	0.09	1–2 *p* = 0.009 1–3 *p* < 0.001 2–3 *p* = 0.021
Dieting	78.62	0.54	0.38	104.43	0.73	0.75	149.41	1.00	0.81	18.18	<0.001	0.08	1–2 *p* = 0.027 1–3 *p* < 0.001 2–3 *p* = 0.011
Bulimia/food preoccupation	76.28	0.00	0.33	106.37	0.33	0.50	139.31	0.50	0.67	17.22	<0.001	0.08	1–2 *p* = 0.005 1–3 *p* < 0.001 2–3 *p* = 0.086
Oral control	93.77	0.14	0.57	102.73	0.29	0.57	119.81	0.43	0.50	2.53	0.283	<0.01	

Note. ED = overall eating disorder score (EAT-26 total); Mdn = median; IQR = interquartile range; H(2) = Kruskal–Wallis H statistic with 2 degrees of freedom; *p* = significance level; η^2^ = effect size. Profile 1 = low internalization and perfectionism; Profile 2 = moderate internalization and perfectionism; Profile 3 = high internalization and perfectionism.

**Table 6:** Mediation models testing the role of maladaptive perfectionism in the relationship between sociocultural internalization and eating disorder risk.

						CI 95%	
Model	Effect ^a^	B	SE	*t*	*p*	LL	UL
Model 1—Internalization—Pressure	a	1.22	0.13	9.09	<0.001	0.96	1.49
	b	0.11	0.02	4.75	<0.001	0.06	0.15
	c	0.34	0.04	7.56	<0.001	0.25	0.44
	c’	0.21	0.05	4.13	<0.001	0.11	0.31
	c–c’	0.13	0.03			0.07	0.20
Model 2—Internalization—Athleticism	a	2.00	0.45	4.47	<0.001	1.12	2.89
	b	0.14	0.02	7.05	<0.001	0.10	0.19
	c	0.58	0.15	3.94	<0.001	0.29	0.87
	c’	0.28	0.14	2.07	0.040	0.01	0.56
	c–c’	0.29	0.08			0.14	0.47
Model 3—Internalization—Information Seeking	a	1.56	0.36	4.27	<0.001	0.84	2.28
	b	0.14	0.02	7.06	<0.001	0.10	0.18
	c	0.48	0.12	4.04	<0.001	0.25	0.71
	c’	0.25	0.11	2.27	0.024	0.03	0.47
	c–c’	0.23	0.07			0.10	0.36
Model 4—Internalization—Information	a	2.08	0.35	5.90	<0.001	1.38	2.78
	b	0.15	0.02	7.08	<0.001	0.11	0.19
	c	0.40	0.12	3.35	0.001	0,17	0.64
	c’	0.08	0.12	0.73	0.464	−0.14	0.32
	c–c’	0.32	0.07			0.19	0.46

Note. The independent variable is indicated for each model (dimensions of sociocultural internalization). In every model, the dependent variable is the severity of eating disorder symptoms. The mediating variable in the models is maladaptive perfectionism. ^a^ c–c’ represents the indirect effect testing mediation; if the confidence intervals do not include the value “0,” the mediation effect is statistically significant at *p* < 0.05.

**Table 7:** Mediation models testing the role of adaptive perfectionism in the relationship between sociocultural internalization and eating disorder risk.

						CI 95%	
Model	Effect ^a^	B	SE	*t*	*p*	LL	UL
Model 1—Internalization—Pressure	a	0.14	0.07	1.97	0.051	−0.01	0.27
	b	0.08	0.05	1.74	0.084	−0.01	0.17
	c	0.34	0.04	7.56	<0.001	0.25	0.44
	c’	0.33	0.04	7.29	<0.001	0.24	0.42
	c–c’	0.01	0.01			−0.01	0.03
Model 2—Internalization—Athleticism	a	0.80	0.20	4.09	<0.001	0.42	1.19
	b	0.08	0.05	1.49	0.138	−0.02	0.18
	c	0.58	0.15	3.94	<0.001	0.29	0.86
	c’	0.51	0.15	3.38	0.001	0.21	0.81
	c–c’	0.06	0.05			−0.03	0.16
Model 3—Internalization—Information Seeking	a	−0.07	0.17	−0.42	0.673	−0.40	0.26
	b	0.13	0.05	2.68	0.008	0.03	0.23
	c	0.48	0.12	4.04	<0.001	0.25	0.71
	c’	0.49	0.12	4.18	<0.001	0.26	0.72
	c–c’	−0.01	0.03			−0.07	0.02
Model 4—Internalization—Information	a	0.15	0.17	0.89	0.376	−0.18	0.47
	b	0.12	0.05	2.31	0.022	0.02	0.22
	c	0.40	0.12	3.35	0.001	0.17	0.64
	c’	0.39	0.12	3.24	0.001	0.15	0.62
	c–c’	0.02	0.02			−0.02	0.06

Note. The independent variable is indicated for each model (dimensions of sociocultural internalization). In every model, the dependent variable is the severity of eating disorder symptoms. The mediating variable in the models is adaptive perfectionism. ^a^ c–c’ represents the indirect effect testing mediation; if the confidence intervals do not include the value “0,” the mediation effect is statistically significant at *p* < 0.05.

## Data Availability

The data are not publicly available due to privacy and ethical restrictions associated with sensitive personal data and participants’ informed consent, but are available from the corresponding author upon reasonable request.
